# Incidental Findings Among Youth Participating in Multimodal Imaging Research: Characteristics of Findings and Description of a Management Approach

**DOI:** 10.3389/fped.2022.875934

**Published:** 2022-06-23

**Authors:** Jessica L. Roane, Megan Mio, Jacqueline Viner, Ariel Bettridge, Chinthaka Heyn, Idan Roifman, Beth Selkirk, Peter Kertes, Bradley J. MacIntosh, Vivekanandan Thayalasuthan, Garry Detzler, Ruby Endre, Laura Jimenez-Juan, Blair Henry, Brian J. Murray, Benjamin I. Goldstein

**Affiliations:** ^1^Sunnybrook Research Institute, Toronto, ON, Canada; ^2^Centre for Youth Bipolar Disorder, Centre for Addiction and Mental Health, Toronto, ON, Canada; ^3^Department of Pharmacology & Toxicology, University of Toronto, Toronto, ON, Canada; ^4^Department of Classics, University of Toronto, Toronto, ON, Canada; ^5^Department of Medical Imaging, University of Toronto, Toronto, ON, Canada; ^6^Department of Medical Imaging, Sunnybrook Health Sciences Centre, Toronto, ON, Canada; ^7^Department of Medicine, University of Toronto, Toronto, ON, Canada; ^8^Department of Medicine, Sunnybrook Health Sciences Centre, Toronto, ON, Canada; ^9^Department of Ophthalmology & Vision Sciences, Sunnybrook Health Sciences Centre, Toronto, ON, Canada; ^10^Department of Ophthalmology & Visions Sciences, University of Toronto, Toronto, ON, Canada; ^11^Department of Medical Biophysics, University of Toronto, Toronto, ON, Canada; ^12^Division of Neurology, Sunnybrook Health Sciences Centre, Toronto, ON, Canada; ^13^Department of Psychiatry, University of Toronto, Toronto, ON, Canada

**Keywords:** youth, bipolar disorder, incidental findings, neuroimaging, imaging

## Abstract

Research imaging in healthy and clinical youth populations yields incidental findings that require a management strategy. Our primary objective was to document the frequency and nature of incidental findings within a research group integrating multiple imaging modalities. A second objective was to describe the evolution of an approach to handling incidental findings. A case example was included to display the intricacies of some of these scenarios. Youth, ages 13–20 years, with bipolar disorder, familial risk for bipolar disorder, or healthy controls, obtained one or a combination of neuroimaging, cardio-thoracic imaging, retinal imaging, and carotid imaging. All images were systematically reviewed for incidental findings. Overall, of 223 participants (*n* = 102 healthy controls), 59% (*n* = 131) had a brain magnetic resonance imaging (MRI) incidental finding and 27% (*n* = 60) had at least one incidental brain finding requiring non-urgent follow-up. In addition, of 109 participants with chest/cardiac MRI and carotid ultrasound, 3% (*n* = 3) had chest findings, 2% (*n* = 2) had cardiac findings, and 1% (*n* = 1) had a carotid finding. Of 165 youth with retinal imaging, 1% (*n* = 2) had incidental findings. While the vast majority of these incidental findings were of a non-serious, non-urgent nature, there were noteworthy exceptions. Imaging research groups need a system that emphasizes the value of clinical review of research images and one that is collaborative and responsive in order to inform follow-up plans. Rating systems that have been developed and used in neuroimaging for the classification of incidental findings can be adapted for use in areas other than the brain. Regardless of severity, incidental findings may raise anxiety in youth participants and their parents. The optimal threshold is one that balances transparency with utility.

## Introduction

The use of imaging in youth research with healthy and clinical populations gives rise to incidental findings that require a management strategy. The prevalence of incidental findings in imaging research, particularly neuroimaging, is well documented across the lifespan (1–5). Whereas studies have reported on incidental findings in clinically indicated imaging of youth (6, 7), little is known regarding incidental findings among youth participating in imaging research in areas other than the brain.

Given the prevalence of incidental findings in neuroimaging, bioethical and medicolegal considerations in this domain have garnered substantial attention in the literature (8–13). To encourage advanced planning, national organizations, institutions, and bioethicists have outlined key considerations and management recommendations (14, 15). Incidental finding management can be guided by principles of autonomy, interests of participants, a researcher's responsibilities/moral obligations, and/or distributive justice (13). A review of ethical considerations involving youth in neuroimaging research outlines numerous themes of concern (16). Despite the robust discourse in the literature, there are no widely accepted recommendations to guide researchers conducting youth imaging studies (17).

In adult neuroimaging research there is variability across studies relating to detection, handling, classification, and communication of incidental findings (8, 10, 14, 18–22). Outside of the neuroimaging community, there is a relative dearth of studies describing the approach to managing the pragmatic aspects of incidental findings. Similarly, the literature as it relates to management of incidental imaging findings in youth is sparse. In addition, despite a high rate of incidental findings in general, concerns have been raised about the risk-benefit balance of having research MRI brain scans read, given the combination of low likelihood of those findings requiring medical intervention alongside concerns about precipitating anxiety (8, 18). The issue of risk-benefit balance is especially relevant for healthy controls (HCs).

Over the past decade, our clinical research group has been conducting imaging studies in the field of youth bipolar disorder (BD), a condition associated with high rates of physical health problems. Participants include youth (ages 13 to 20) with BD, youth offspring who have a parent or sibling with BD, as well as HCs. Our studies have examined neuroimaging, cardio-thoracic imaging, retinal imaging, and carotid imaging. During the course of managing incidental findings, many questions arose for which the literature did not offer definitive direction. We came to learn that other groups have raised very similar questions (14, 23). For example, do all images need to be reviewed? If so, by whom? How are incidental findings categorized? How are incidental findings in areas other than the brain managed? Are all incidental findings conveyed to the participants? Who is responsible for arranging the follow-up? How should incidental findings be conveyed and by whom? Should both the youth and the parents be informed? This article will describe *the evolution of an approach to handling incidental findings and the ways in which the aforementioned questions have been addressed*, along with descriptive data regarding the frequency and nature of incidental findings. A case example is included to demonstrate the complexities of some of these scenarios.

## Method

### Participants

Participants with BD and those who have a parent or sibling with BD were recruited from a clinical-research program focused on youth BD, based in a tertiary general hospital; HC participants were recruited from community advertisements. All participants were enrolled in studies that included imaging protocols. All studies were approved by the local research ethics board. Prior to any procedures, written informed consent was obtained from both participants and a parent or guardian. Exclusion criteria were determined by the individual study protocols.

### Brain Magnetic Resonance Imaging

Images of the brain were acquired using a 3 Tesla (3T) Philips Achieva System with an 8-channel head-receiver coil or a 3T Siemens Prisma using a 20-channel head-neck coil. Structural T1-weighted images and T2-weighted fluid attenuated inversion recovery (FLAIR) images were reviewed by a staff neuroradiologist.

### Cardio-Thoracic Magnetic Resonance Imaging

To obtain cardio-thoracic images, participants were positioned in the 3T Siemens Prisma MRI and a 16- or 32-channel cardiac phased array coil was used on chest, at the level of the heart; two elements of a spine array coil were used as posterior coil elements. Consecutive transverse and oblique sagittal slices of the thoracic aorta were obtained with a standard spin-echo sequence. Participant variation existed with regards to anatomical visibility due to the distal image acquisition. In all images the heart, aorta, liver, and lungs were visible. The spine, spleen, kidney, pancreas, lymph nodes, and thyroid gland may also have been visible. A cardiologist with a level 3 certification in cardiac MRI by the Society for Cardiovascular Magnetic Resonance reviewed all cardiac images, and a cardiothoracic radiologist reviewed all extra-cardiac images.

### Retinal Photography

Retinal fundus images were collected by a certified ophthalmic assistant (B.S.) using a Topcon TRC 50 DX, Type 1A camera following pupil dilation with 1% tropicamide and 2.5% phenylephrine eye drops. Images were taken at a 50-degree angle and captured the optic disc, macula, and retinal microvessels. Participants were asked to refrain from consuming any products containing caffeine or nicotine as both substances elicit vasoactive effects and may bias retinal photography measurements. ImageJ Software (National Institutes of Health, Bethesda, Maryland, USA) was used to compute vessel diameters.

### Carotid Ultrasound

High-resolution B-mode carotid ultrasound scanning was performed with an ultrasound machine optimized for carotid imaging. A trained sonographer measured combined thickness of intima and media of the far wall of both common carotid arteries. The distance between lumen-intima and media-adventitia boundaries was also measured. The carotid images were reviewed by an imaging specialist, with escalation, if needed, to a collaborating cardiothoracic radiologist with expertise in carotid imaging.

### Incidental Finding Classification

**Figure 1:**
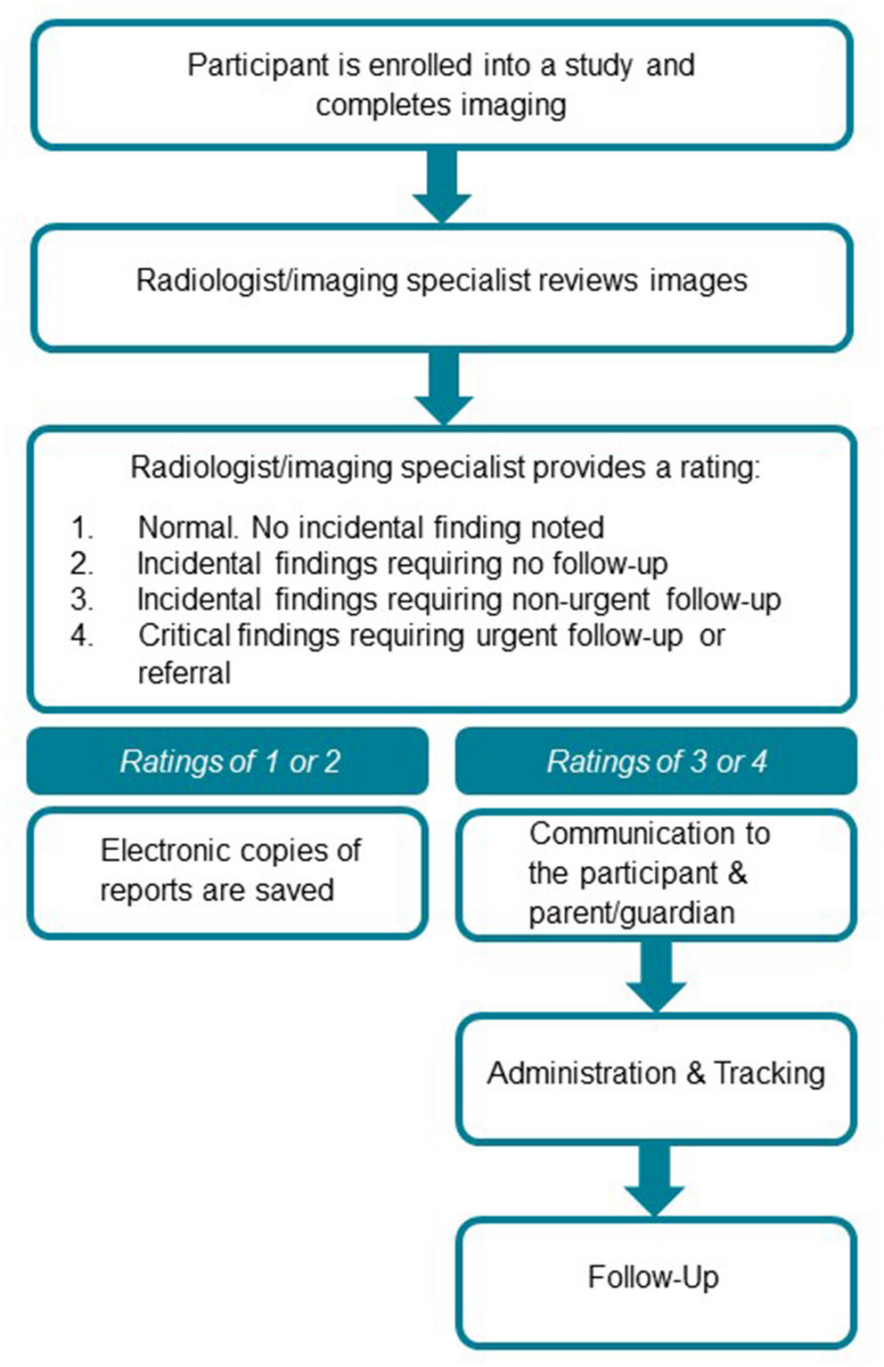
Incidental findings management pipeline and workflow.

Classification of neuroimaging incidental findings was based on the following 4-point rating system: 1- normal, no incidental finding; 2 - incidental finding requiring no follow-up; 3 - incidental finding requiring non-urgent follow-up; 4 - critical finding requiring urgent follow-up or referral (Figure 1). This scoring convention was derived from an established rating scale (3). A similar 4-point rating scale has also been used with a population-based pediatric sample (5). In accordance with institutional neuroradiology practices, sinus findings were also reported when reviewing scans. This scoring system was also used by our collaborating cardiologist and cardiothoracic radiologist. Of note, several incidental findings were described as provisional/suspected, including those that required follow-up patient visits and investigations for definitive confirmation. Throughout this manuscript, we include all suspected/provisional findings as incidental findings.

Our lab manager provided the imaging specialists with scans to be read and obtained the reports once they were rated. No subsequent action was taken for scores of 1 or 2. For all reports that were rated as a 3 or 4, the lab manager and research coordinator met to verify the accurate identity of the participant. The principal investigator (PI), lab manager, and research coordinator met to review the abnormal scan reports. The PI determined the appropriate follow-up based on the recommendation of the specialist, and the preference of the research participant, and proceeded with one of the following: (1) ordered follow-up investigation at our institution; (2) recommended follow-up with the primary care physician (PCP); or (3) provided the participant with the choice to follow-up at our institution or with their PCP. The PI provided the research coordinator with language to communicate the incidental finding to the participant.

The process for the review of retinal images was that the ophthalmic photographer brought any potential abnormality to the attention of the primary collaborating ophthalmologist, a retina specialist. Any clinically relevant finding was communicated directly to the PI. Because incidental retinal findings were rare and reviewed in real time, the aforementioned rating system was not used.

### Communication to the Participant and Parent/Guardian

The following approach evolved over time based on experience. If the research participant was under clinical care of the PI, the PI generally opted to communicate the incidental finding within the context of a medical appointment. For other participants, the research coordinator called to communicate the incidental finding, confirmed the follow-up plan, and obtained permission to share all information with the PCP. When appropriate, the research coordinator offered that a copy of the scan be provided on a disc in order to facilitate clinical follow-up outside of our institution. At least 3 attempts were made to communicate the result directly to the youth. While the consent form did not specify such a circumstance, if the youth could not be reached, the decision was made to communicate the result to a parent/guardian. The parent/guardian was asked if they were comfortable sharing the finding with their youth. We requested that the parent/guardian ask the youth to call the research coordinator so that information could be conveyed to them directly and so that there was an opportunity to ask questions. If the youth was initially contacted, the research coordinator asked if they were comfortable sharing the finding with their parent/guardian, or if they preferred that the research coordinator did so directly.

### Administration and Tracking

All contact attempts and conversations were documented on a communication log. A letter was drafted and faxed to the PCP, including details regarding the plan for follow-up, or that highlighted the recommendation for outstanding follow-up. A copy of the imaging report was faxed to the PCP. The following documentation was filed: MRI report, letter to doctor, fax transmission report, communication log and follow-up requisition if needed.

### Follow-Up

The research coordinator followed-up with the participant and/or parent after 1 month to confirm they followed the guidance provided and tracked completion of scheduled follow-up appointments. The PI, lab manager, and research coordinator met monthly to review all outstanding incidental findings and this management approach.

### Statistical Analyses

To assess for demographic and clinical differences in participants with vs. without incidental findings, independent-sample *t*-tests or Mann-Whitney *U*-tests were employed for continuous variables and χ^2^ tests for categorical variables. Normality of continuous variables was confirmed using the Shapiro-Wilks test. We opted to examine group differences in diagnosis, age, sex, and race between those with and without brain MRI incidental findings. Given that a number of participants have completed multiple research-related MRIs, in order to avoid double counting incidental findings we opted to report only the first scan upon which an incidental finding was observed. Due to the relatively small number of cardiac, chest, retinal, and carotid incidental findings, as well as the low number of participants with multi-system findings, we did not analyze these cases statistically but rather reported these findings descriptively as rates or case examples. All statistical analyses were conducted with Statistical Package for the Social Sciences (SPSS), version 25.0.

## Results

The following section outlines the incidental findings according to location.

### Brain Magnetic Resonance Imaging

**Figure 2:**
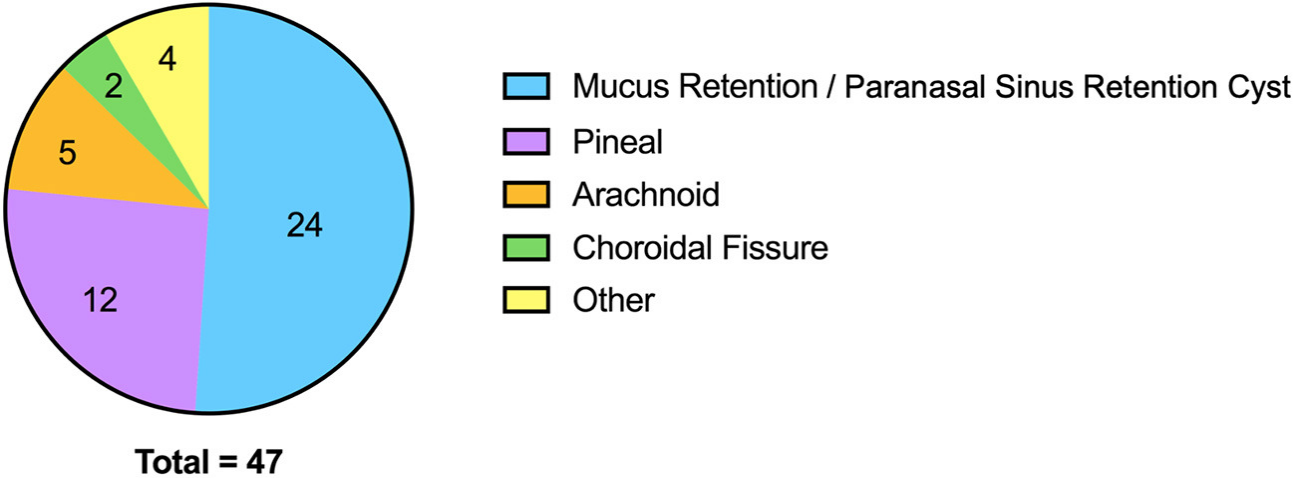
Breakdown of cyst incidental findings on brain MRI scans. “Other” includes subependymal, Tornwaldt, posterior sella, dermoid (*n* = 1 for each).

Overall the cohort included 223 youth (mean age of 17.04 ± 1.62 years); *N* = 97 BD, 102 HC, and 24 youth at familial-risk of BD. The cohort was 57% female (*N* = 128 female, 95 male) and 67% Caucasian (*N* = 150 Caucasian, *N* = 73 non-Caucasian). The frequencies of brain MRI incidental findings are reported in Table 1. Of 223 individuals, incidental findings were reported in at least one scan for 59% of individuals (*n* = 131). The most common incidental finding was the presence of white matter hyperintensities (WMH) of presumed vascular origin (34%), followed by benign cysts (19%), and reactive sinus changes/inflammation (19%). A breakdown of cyst incidental findings on brain MRI scans is seen in Figure 2. Male sex [χ^2^(1, *N* = 223) = 6.40, *p* = 0.01] and Caucasian race [χ^2^(1, *N* = 223) = 3.98, *p* = 0.046] were both significantly associated with the presence of any brain MRI incidental finding. There were no significant differences in the frequency of overall brain MRI findings based on age (t = 1.66, *p* = 0.10) or participants with vs. without BD [χ^2^(1, *N* = 223) = 0.10, *p* = 0.75]. Caucasian race [χ^2^(1, *N* = 223) = 8.53, *p* = 0.03] was associated with the presence of cysts.

**Table 1:** Frequency of specific types of incidental neuroimaging findings.

**Brain MRI incidental**	**Number of individuals** **(% Total)**
White matter hyperintensities	75^*^ (34)
Benign cysts	43 (19)
Reactive sinus changes and inflammation	43 (19)
Developmental venous anomaly	11 (4.9)
Benign anatomical abnormalities	8 (3.6)
Benign tumors or nodules	6 (2.7)
Low lying cerebellar tonsils	4 (1.8)
Infarct	3 (1.3)
Diffuse cerebral/cerebellar volume loss or atrophy	3 (1.3)
Prominence of anterior communicating artery	2 (0.9)
Enlarged perivascular spaces	2 (0.9)
Cavum septum pellucidum	2 (0.9)
Incidentals with 1 case (<0.5%):	
• Aneurysm; canalis basilaris medianus; encephalomalacia; frontal cortical dysplasia; hemangioma; lesions indicating demyelination; non-aggressive osseous lesion; petrous effusion; pituitary adenoma; prominent diploic veins	

* *Given the high frequency of WMH, we also report here the related classification scores: Level 2 (no follow-up), n = 37; Level 3 (non-urgent follow-up), n = 37; Level 4 (urgent follow-up), n = 1*.

Out of 223 individuals, 41% (*n* = 92) were categorized as normal, 31% (*n* = 68) had at least one incidental finding requiring no follow-up, 27% (*n* = 60) had at least one incidental finding requiring non-urgent follow-up, and 1% (*n* = 3) had at least one scan that was a critical finding, requiring urgent follow-up (Table 2). The following 3 abnormalities required urgent follow-up: (1) white matter lesions with appearances that were concerning for demyelination; subsequent MR with gadolinium contrast was completed followed by referral to neurology; (2) suspected 3mm cerebral aneurysm; subsequent confirmatory MR angiography was completed followed by referral to neurosurgery; (3) 16 mm pineal cyst causing mild mass effect on the midbrain tectum; subsequent MR with gadolinium contrast was obtained, followed by referral to neurosurgery.

**Table 2:** Frequency of incidental finding classification scores.

**Incidental finding classification**	N^*^(%)
1–Normal, no incidental	92 (41)
2–Incidental, no follow-up	68 (31)
3–Incidental, non-urgent follow-up	60 (27)
4–Critical finding, urgent follow-up	3 (1)

* *For simplicity, findings are presented at the individual level. For participants with more than one incidental finding, within this table they were classified according to their most severe incidental finding*.

### Thoracic Magnetic Resonance Imaging

A total of 109 participants underwent cardiothoracic MRI, of which abnormalities were detected in 3 (3%) participants. Abnormalities included a splenic hemangioma/cyst, a high intensity hepatic lesion, and a renal cortical cyst. Two (2%) of these MRI incidentals required non-urgent follow-up and 1 (1%) did not require follow-up.

### Cardiac Magnetic Resonance Imaging

Incidental findings were noted for 2 of 109 participants who completed cardiac MRI (2%). Both required non-urgent follow-up. For one participant, the finding was a D-shaped septum indicative of potential right ventricular pressure overload, possibly due to pulmonary hypertension. Another participant had a left ventricular dilatation and hypertrabeculation, with normal left ventricular function, thought to possibly reflect early idiopathic non-compaction of the myocardium. Subsequent investigation revealed that this patient lacked the diagnostic criteria required for such a diagnosis and that the findings likely reflected normal variability.

### Retinal Photography

Incidental findings were noted for 2 of 165 participants who completed retinal photography (1%). One participant had retinal pigment epithelium changes temporal to the fovea. This finding was described as non-urgent and the participant was seen in follow-up by our collaborating ophthalmologist, a retina specialist. In another participant, there was grouped pigmentation of the retinal pigment epithelium, of no visual consequence, that can be associated with Gardner's syndrome. In this case, the participant was already aware of this abnormality.

### Carotid Ultrasound

A total of 109 participants completed carotid artery ultrasound. Only one abnormality was detected across ultrasounds (1%). The participant had bilateral enlarged lymph nodes and this finding was also observed on MRI where it corresponded with mucosal thickening of paranasal sinuses. This case required urgent follow-up.

### Multimodal Imaging

Incidental findings were documented across a number of research studies and imaging modalities and therefore the total number of participants who have completed a particular scan vary. Brain MRI incidentals were reported in 59% (131/223), chest MRI incidentals in 3% (3/109), cardiac MRI incidentals in 2% (2/109), retinal incidentals in 1% (2/165), and carotid MRI incidentals in 1% (1/109). Five participants had incidental findings in two systems: potential pulmonary hypertension detected via cardiac MRI plus a brain MRI finding of an enlarged pituitary; left ventricular dilatation/hypertrabeculation detected via cardiac MRI plus numerous cysts reported on brain MRI; splenic hemangioma/cyst detected via cardiac MRI plus suspected chronic infarct detected via brain MRI; renal cortical cyst detected via cardiac MRI plus arachnoid cyst detected via brain MRI; enlarged bilateral lymph nodes detected through carotid ultrasound and brain MRI findings of linear nodule in frontal white matter, enlarged bilateral lymph nodes, and mucosal thickening in paranasal sinuses.

### Case Example

Jennifer (not her real name) is a 19 year old female with BD, anxiety, and attention deficit hyperactivity disorder who participated in imaging research studies with our group. Jennifer had incidental research findings in two systems (brain and abdomen), with additional findings identified in the context of clinical work-up of the research scans (cardiac and aorta). A series of unlikely events culminated in an emergency department (ED) visit for suspected aortic dissection, with subsequent exacerbation of anxiety symptoms. This case is an example of potential unintended consequences of radiological review of research imaging in youth as well as the potential benefits (i.e. early detection with opportunity for preventive intervention) of review and follow-up of incidental findings. The full clinical details along with a first-person narrative are provided as Supplementary Material.

## Discussion

This article describes a process that evolved with experience and depicts the way in which our clinical research group came to approach the detection, classification, communication, and management of incidental findings. While we have not evaluated the relative strengths and limitations of our system in comparison to alternatives, we have described it here to inform the approaches of other imaging teams. In addition to sharing practical details, below we have provided anecdotal observations, and descriptions of the ways in which we have answered the fundamental questions posed at the beginning of this manuscript.

In our sample, the rate of reported incidental brain MRI findings was over 50%, which is approximately three times higher than reported in a recent meta-analysis (2). There are a number of possible explanations for the variability in the literature and our comparatively higher reported rates of incidental findings. First, there are differences between neuroradiologists in regards to thresholds for characterizing an imaging feature as an incidental finding. Second, the variability in the prevalence of incidental findings on research scans is influenced by the imaging sequences that are performed. Our studies acquired high resolution T1 and T2 FLAIR images, which facilitate incidental findings detection compared to standard resolution sequences. In particular, T2 FLAIR images increase sensitivity to detection of WMH, which represented the most common incidental finding in our study. While often excluded from the prevalence of incidental findings reported in adults, WMH are especially important in the young population under investigation and such abnormalities often require careful radiologist assessment (4). Finally, many neuroimaging studies do not evaluate/report sinus disease, whereas the neuroradiologists who reviewed participant scans in our study did report on sinus disease.

In addition to neuroimaging, 1–3% had incidental findings in chest MRI, cardiac MRI, retinal imaging, and/or carotid ultrasound. These findings can begin to fill the gap in knowledge regarding incidental findings outside of the brain among youth participating in imaging research.

In 2017 the research institute and research ethics board at the study site released guidelines for the management of incidental findings in imaging studies, including the instruction that follow-up be arranged by the investigators and not deferred to external physicians. With the introduction of these guidelines, our institution provided a local answer regarding *who is responsible for arranging follow-up*. Prior to that guideline, the informed consent process was not consistently explicit about the review and reporting process and follow-up in HC participants was often deferred to the PCP. The sociocultural context of research populations, including access to a PCP is an important consideration in the development of incidental findings protocols (24). The consent process should include explicit information on screening, the threshold for reporting, and management of incidental findings (23). Additional key recommendations from the ethics literature on the consent process have been summarized (16).

Receiving an unexpected phone call communicating an incidental finding can evoke fear for young research participants and their parents. One might have predicted that these phone calls would be especially difficult for youth with BD, most of whom also have anxiety disorders. Although we did not systematically follow-up after incidental finding calls and it is possible that youth did experience some emotional dysregulation, the majority of youth with BD were under our psychiatric care and no incidents of destabilization or significant dysregulation came to the team's attention. We speculate that this is at least partly related to the fact that these youth have acquired a degree of resilience through the management of their chronic mental health symptoms and related stressors. Alternatively, participants, most of whom were patients of our institution, had developed comfort and trust in our group and the organization. Although the incidental findings themselves were triggered by participation in research, follow-up often became integrated with their pre-existing psychiatric care. Compared to the BD participants, the HC participants generally expressed more surprise and distress upon receiving a call regarding an incidental finding. Relatedly, while we expected higher rates of WMH in youth with BD vs. HCs, we did not observe such a difference, as articulated in detail in a prior publication focused on WMH specifically (25).

In most cases, particularly with younger participants, when asked if they were comfortable sharing the information with their parent, or if they preferred that the research coordinator call them directly, they opted for the research coordinator to make contact. Parents often conveyed more concern than participants. In several instances, the parents were surprised and angry that their youth's findings had first been communicated to the youth. Conversely, in cases when the youth could not be reached, necessitating the communication of their incidental finding to a parent first, the youth generally did not voice concern that this had been done. Our group approached this challenge by balancing efforts to communicate the finding in a timely manner, with sharing the finding directly to participants first. In the province of Ontario, consent is based on capacity with regard to medical decision making, rather than age. In some cases when the finding was shared with the parent first, the parent did not wish to share the finding with their youth or preferred to defer this for personal reasons. In these cases the conclusion was reached to accept the limits of our influence. We did not consider or encounter the potential scenario of a competent youth wishing to know about a non-urgent finding and the parent does not. A capacity adjusted framework for decision making about incidental findings in neuroimaging can be used to guide these decisions around disclosure (16). In addressing the question, *should both the youth and the parents be informed*, we found that it is reasonable to attempt to involve both parties, with an increased emphasis toward parental involvement with younger youth. A discussion regarding who will be informed of incidental findings should be included in the informed consent process.

As our system evolved and the number of incidental findings increased, the PI enlisted assistance from MSW-level social workers/research coordinators. This represents the most significant divergence in our approach compared to those described in the literature and provides an alternative answer to the question regarding *how incidental findings should be conveyed and by whom*. Considerations were made in order to communicate the finding with clear and simple language in a manner that could ease participant anxiety whenever possible, but that is sufficiently detailed to convey importance. While participants had the option for further discussion with the PI, this was rarely requested. In our experience, the involvement of additional staff allowed the research team to play a more involved role in ensuring that participants did not “fall through the cracks” and providing facilitation to support participants in following clinical recommendations.

Prior to our implemented system for managing incidental findings, on occasion an MRI technician would flag a suspected abnormality during a scan and that would necessitate a subsequent read by a neuroradiologist. Of note, the three cases previously reviewed that required urgent follow-up were not flagged by the MRI technician. This underscores recommendations that have been made previously regarding the value of having radiological review for all imaging scans. Given the high rates of incidental findings that were ultimately deemed not clinically actionable, it is likely that we will increase the threshold for conveying incidental findings in future studies, balancing transparency with utility. Our research group has also moved toward providing participants with the choice to be informed of potentially clinically relevant incidental findings, or not.

We were able to develop collaborations with imaging specialists, and establish pipelines for systematic review of research images. A previously published rating system for incidental neuroimaging findings was readily adapted for use with other domains (e.g., cardiac). Collectively, these findings can be used to inform participants during the informed consent process and contribute to discussions of potential risk and benefits of participating. This subject requires collaboration of researchers, radiologists, research ethics boards, clinical ethicists, and research participants in order to appropriately identify and manage incidental findings requiring follow-up. A responsible research program should endeavor to address these factors in an organized and supportive manner, with an emphasis on continuous self-improvement informed by participant input. Finally, empirical studies are needed to evaluate and compare different incidental finding systems, and such studies should integrate measures of clinical, system-related, and experiential outcomes.

## Data Availability Statement

The raw data supporting the conclusions of this article will be made available by the authors, without undue reservation.

## Ethics Statement

The studies involving human participants were reviewed and approved by the work described in this manuscript has been carried out in accordance with the Code of the World Medical Association (Declaration of Helsinki). Consent was obtained from all participants and their parent and/or guardian prior to participating. This study used data from a number of studies. Ethical approval was granted by Sunnybrook Research Institute Research Ethics Board for all studies from which data was used. REB: 409-2013, 408-2011, 405-2014, 435-2015, and 008-2018. All data was collected at Sunnybrook Research Institute. However all data was transferred with the Center for Youth Bipolar Disorder's relocation to the Center for Addiction and Mental Health. Thus, ethical approval was also granted by CAMH Research Ethics Boards. REB: 165/2020, 168/2020, 152/2020, 163/2020, and 173/2020. Written informed consent to participate in this study was provided by the participants' legal guardian/next of kin. Written informed consent was obtained from the individual(s) for the publication of any potentially identifiable images or data included in this article.

## Author Contributions

JR primarily wrote the manuscript and was responsible for the communication of incidental findings to participants. MM performed statistical analyses and provided all figures. JV wrote a section of the manuscript. AB contributed to the incidental finding management approach. CH reviewed all brain magnetic resonance imaging scans. IR reviewed all cardiac images. BS performed all retinal photography and PK reviewed incidental retinal findings. BMa contributed to study conception and design. VT reviewed carotid images. GD and RE conducted all brain MRI scans. LJ-J reviewed all extra-cardiac images. BH and BMu provided consultation regarding ethical issues associated with incidental findings. BG contributed to study conception, design, and assisted with manuscript preparation. All authors contributed to manuscript revisions, read, and approved the submitted version.

## Funding

The research was supported by the Canadian Institutes of Health Research under grants (PJT 162110 and MOP 136947), the Heart and Stroke Foundation under grant (G-17-0017597), and the SickKids Foundation under grant (NI18-1295).

## Conflict of Interest

PK declares his position on the advisory boards for Novartis, Alcon, Bayer, Novelty Nobility; Institutional financial support from Allergan, Bayer, Roche, Novartis; Personal financial support from Novartis, Bayer; and his position as an equity owner for ArcticDx. The remaining authors declare that the research was conducted in the absence of any commercial or financial relationships that could be construed as a potential conflict of interest.

## Publisher's Note

All claims expressed in this article are solely those of the authors and do not necessarily represent those of their affiliated organizations, or those of the publisher, the editors and the reviewers. Any product that may be evaluated in this article, or claim that may be made by its manufacturer, is not guaranteed or endorsed by the publisher.
