# Supplementary figures and images for: Incidental Findings Among Youth Participating in Multimodal Imaging Research: Characteristics of Findings and Description of a Management Approach
Source: Front Pediatr. 2022 Jun 23;10:875934. doi: 10.3389/fped.2022.875934 (PMC9259791; doi:10.3389/fped.2022.875934)

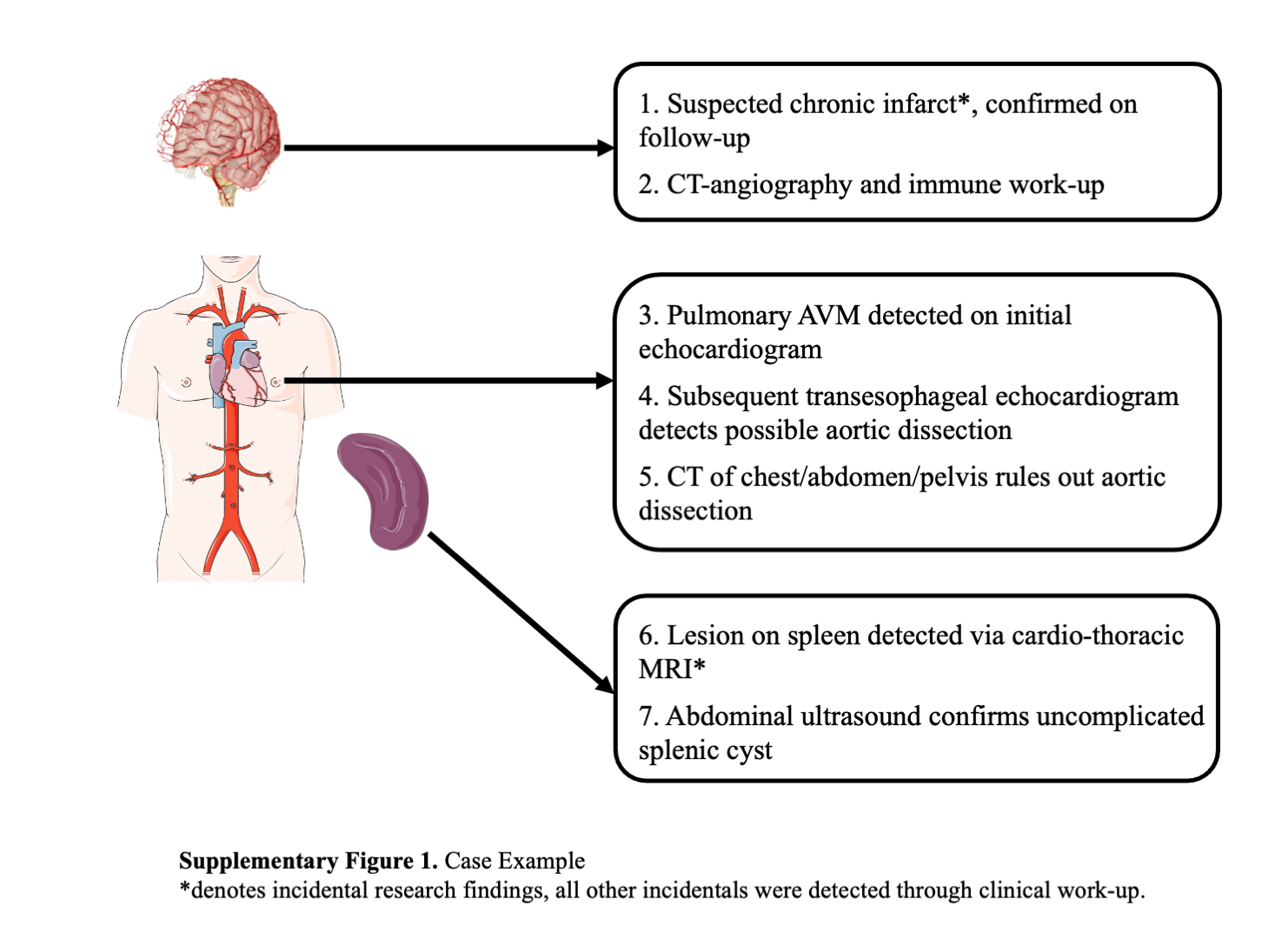

Supplement: Supplementary file 1 [file Image_1.TIFF]
