# Supplementary material for: Incidental Findings Among Youth Participating in Multimodal Imaging Research: Characteristics of Findings and Description of a Management Approach
Source: Front Pediatr. 2022 Jun 23;10:875934. doi: 10.3389/fped.2022.875934 (PMC9259791; doi:10.3389/fped.2022.875934)
Supplement: Supplementary file 2 [file Table_1.DOCX]

**Case Example**

Jennifer (not her real name) is a 19 year old female with BD, anxiety, and attention deficit hyperactivity disorder who participated in a neuroimaging research study with our group. That study identified incidental findings that through a series of unlikely events ultimately led to an emergency room visit for suspected aortic dissection. This case is an example of potential unintended consequences of radiological review of research imaging in youth. The cascade of follow-up imaging and tests is seen in supplementary figure 1.

On Jennifer’s research brain MRI, WMH were noted, along with an incidental finding of suspected chronic infarct. The neuroradiologist suggested a diagnostic clinical MRI and a referral to stroke neurology. The diagnostic MRI confirmed the appearance of old infarcts corresponding to the left middle cerebral artery territory; previous infection and vasculitis were included within the differential diagnosis. Jennifer saw a stroke neurologist who ordered a computed tomography (CT) angiography, and echocardiography with bubble study, along with blood work to rule out autoimmune conditions or a hypercoagulable syndrome. Jennifer’s CT angiography and autoimmune workup were unremarkable. Her echocardiogram demonstrated late bubble crossover, interpreted as more consistent with pulmonary arteriovenous malformation rather than intracardiac shunt. This finding led to a subsequent transesophageal echocardiogram to further evaluate for a pulmonary arteriovenous malformation. During the transesophageal echocardiogram an echodensity was noted in the descending aorta which was suspicious for aortic dissection, prompting a referral to the emergency department (ED). Jennifer was seen by the ED physician and referred to cardiac surgery. The prospect of cardiovascular surgery was discussed with Jennifer and her parents, and she received a labetolol infusion to reduce systolic blood pressure, and two large-bore intravenous lines were placed in preparation for potential cardiovascular surgery. Ultimately, CT chest/abdomen/pelvis ruled out aortic dissection or any other obvious abnormality and Jennifer was cleared by cardiology and vascular surgery staff and was discharged home with a diagnosis of “chest pain not yet diagnosed”. In the days after her ED visit, Jennifer experienced chest pain radiating into her arm and returned to the ED where she was cleared. In retrospect she was able to attribute this to anxiety. While Jennifer did not at any point meet criteria for post-traumatic stress disorder, there was evidence of increased anxiety in general, and hyperarousal in particular, in the months following these events.

Six months after Jennifer’s initial research participation, she participated in a subsequent research study and this time obtained cardio-thoracic imaging which identified a 1.5 cm high-intensity lesion in the lower pole of the spleen, favored benign in nature, likely a hemangioma or cyst. Non-urgent abdominal ultrasound was recommended. The abdominal ultrasound was obtained and confirmed a probable complicated cyst at the lower pole of the spleen. Reassessment with ultrasound in 6-12 months in order to ensure stability was recommended.

**Personal Narrative Regarding Case Description**

The following are several questions posed to Jennifer regarding her experiences, along with her answers.

**Had you given thought to the possibility of an incidental finding?**

*“I had never really given thought to the possibility of an incidental finding before participating in research. In the back of my mind I was pleased to be getting an MRI just to make sure everything was okay. I guess I just thought everything would be okay. Right now things are okay. When you have bipolar disorder, however, ‘right now’ isn’t that comforting.”*

**What was your first reaction to hearing about the finding?**

*“The words ‘white matter’ didn’t mean anything to me at the time. I remember thinking something along the lines of: ‘oh, of course this is happening.’ This has been my mentality throughout this whole thing. In many ways it had to be. Over the years I have learned that if your mental health is laughable, it’s okay to just laugh sometimes. Why should my physical health be any different? It’s not denial or complacency. It comes from a place of realizing that I am more than just my mind and body - a realization I was forced to have way too young subsequent to my bipolar disorder diagnosis. Even if my mind and body fail me sometimes, there is so much more left of me to lose - like my sense of humor. I do admit it is hard sometimes.”*

**Looking back on the whole process, what do you think of how things were handled?**

*“I am very grateful for all the help I have received. I take care not to blame the mystery of my predicament on the people who are doing their best to help me through it all.”*

**What were the better and worse aspects of the process?**

*“After the ED ordeal, the doctor I was referred to described my experience as being on a medical rollercoaster. Sometimes, he explained, you just need to get off the ride. After a while, all of the tests and the follow-ups and the anxiety just leave you with the question of ‘what are we really doing this for?’ Even if there is an answer out there, is it really going to change anything? Getting off the ride seemed like the best decision. When it comes to your health, however, there is no return to ignorance. Even though I am no longer being regularly confronted with empirical evidence of my bad habits - drinking and smoking, for example - the worst part has been adopting this medical gaze without really wanting to make different choices. Does the possibility of dropping dead in my 20s sometimes rekindle a desire to rebel against my own body? Yes, but I’ll work through it again.”*

**Has this impacted your view of imaging research? If so, how?**

*“This experience hasn’t impacted my view of imaging research at all. Things happen and it’s important work. In many ways I’m grateful for this experience and I’m happy knowing that there was a greater good involved.”*

**What advice to you have for other researchers doing this type of research?**

*“Throughout my medical rollercoaster ride I have felt very supported by those who conducted the research. I got really lucky in that way. It wasn’t just thrown at me and then I was shipped off to another floor of the hospital. Empathy, if you can afford it, goes a long way. No matter what you may find, every brain belongs to someone. It’s more than just an image.”*

**What advice to you have for youth and parents?**

*“Don’t be afraid to participate in research because it really is important. The fear of an incidental finding, I believe, has less to do with the research and more to do with the fear of something being wrong with us. If you know that something is wrong at least you can begin to fix it. If you believe in the work then you shouldn’t let it stop you. My advice is to cross that road if or when you get to it.”*
